# The Effect of the ‘Touch Screen-Based Cognitive Training’ for Children with Severe Cognitive Impairment in Special Education

**DOI:** 10.3390/children8121205

**Published:** 2021-12-19

**Authors:** In Young Sung, Jin Sook Yuk, Dae-Hyun Jang, Gijeong Yun, Chunye Kim, Eun Jae Ko

**Affiliations:** 1 Department of Rehabilitation Medicine, Asan Medical Center, University of Ulsan College of Medicine, Seoul 05505, Korea; iysung56@gmail.com; 2 Department of Rehabilitation Medicine, Asan Medical Center, Seoul 05505, Korea; aosoon@naver.com; 3 Department of Rehabilitation, Incheon St. Mary’s Hospital, College of Medicine, The Catholic University of Korea, Incheon 21431, Korea; dhjangmd@naver.com; 4 Department of Rehabilitation Medicine, Yunsan Gwanjajae Geriatric Hospital, Pusan 47570, Korea; nisus82@naver.com; 5 Seoul Jungjin Special School, Seoul 08259, Korea; kcy3354@naver.com

**Keywords:** touch screen-based cognitive training, education of intellectually disabled, special education, cognitive impairment, child

## Abstract

Traditional education in special schools have some limitations. We aimed to investigate if the ‘touch screen-based cognitive training’ is feasible and effective for children with severe cognitive impairment (developmental age 18–36 months) in special education. In this case, 29 children were randomly allocated to intervention (*n* = 17, ‘touch screen-based cognitive training’, 30 min/session, 3 times/week, 12 weeks) and control (*n* = 12, traditional education) groups. Psychoeducational Profile-Revised (PEP-R), Early Childhood Behavior Questionnaire (ECBQ), Sequenced Language Scale for Infants (SELSI), Pediatric Evaluation of Disability Inventory (PEDI), and Goal Attainment Scale (GAS) were measured before and after 12 weeks of education. The ‘touch screen-based cognitive training’ was applicable in special education. When repeated measures analysis of variance (ANOVA) was used, significant groupⅹtime effect was found for GAS, and significant group effect was found for ECBQ (attentional shifting) and GAS. When adjusting for pre-education measurements, the intervention had a significant effect on the post-education measurements of ECBQ (attentional shifting) and GAS (*p* < 0.05). No relationship existed between the degree of improvements and the severeness of developmental delay in the measurements. ‘Touch screen-based cognitive training’ in special school was feasible and it improved cognition in children with severe cognitive impairment (developmental age 18–36 months), irrespective of the severeness of the developmental delay.

## 1. Introduction

Cognitive impairment is an important issue in children, which is accompanied by cerebral palsy, intellectual disability (ID), autism spectrum disorder (ASD), or other genetic syndromes. Cognitive function affects communication, social participation, and learning skills. These may eventually affect activities of daily living (ADL), education, and social participation [1]. Therefore, cognitive training of such children is important.

Children with cognitive impairment need special education in special schools. Generally, they are taught by teachers. However, sometimes these children have poor motivation to do well in school and poor attention, so that they cannot actively participate in class activities, leading to lower academic achievement [2]. Furthermore, there are usually many children assigned to one teacher (more than 4 children per teacher in Korea) and the degree of learning difficulty is different among the children. Therefore, the teachers cannot always provide individualized special education. Thus, there are some limitations to the existing traditional education in special schools. Furthermore, in the coronavirus disease 2019 (COVID-19) era, close personal contacts with other people are limited. Therefore, an alternative form of education, in which direct personal contacts between children and special education teachers are decreased, is needed.

The ‘touch screen-based cognitive training’ was developed for young children or people with severe cognitive impairment (cognitive age < 4 years) in 2013 [3]. This is the first intervention targeting children with aforementioned characteristics that uses a tablet computer, which enhances convenience for children with both motor and cognitive impairments. It uses animations and sounds that increase children’s attention. There was a previous study showing the feasibility and efficacy of improving cognition through the intervention in a hospital setting [4]. Since it has advantages in standardization, objective feedback, and portability, this intervention may also benefit the children in special education.

Therefore, this study aims to evaluate if the ‘touch screen-based cognitive training’ is feasible and effective for children with severe cognitive impairment in special education.

## 2. Materials and Methods

### 2.1. Study Design and Participants

It was a pilot study and was approved by the ethical committee of Asan Medical Center (reference number: 2018-1539). The study was registered at Clinical Research Information Service (reference number: KCT0003551). 

**Figure 1:**
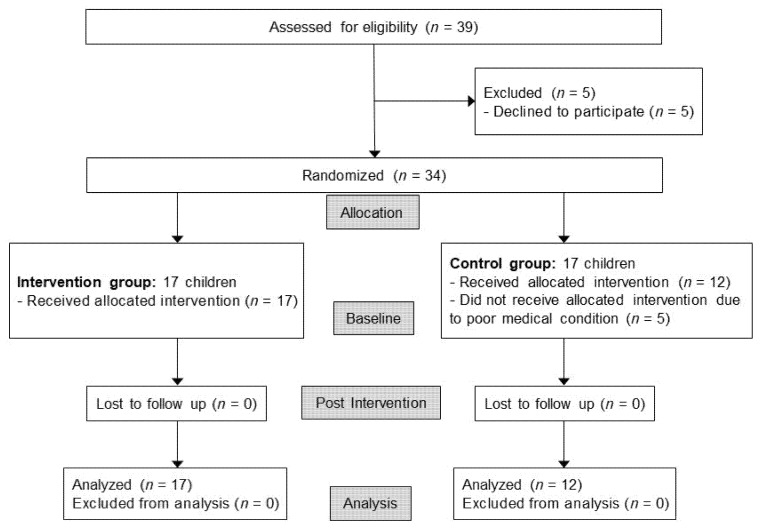
Flow chart.

Children who visited the Pediatric Rehabilitation Medicine Division at Asan Medical Center from January 2014 to December 2016 were assessed for eligibility. The inclusion criteria were as follows: (1) children who went to special schools during this period, (2) children with severe cognitive impairment (cognitive age 18–36 months), as assessed by the developmental age of the Psychoeducational Profile-Revised (PEP-R), and (3) children with a written informed consent from the main caregivers. Exclusion criteria were children with severe motor or visual impairment who cannot participate the intervention. A total of 34 children from 9 special-education schools were enrolled, but 5 could not receive allocated intervention due to poor medical condition (Figure 1). 

### 2.2. Randomization and Blinding

Children were randomly allocated to intervention group (a ‘touch screen-based cognitive training’ as an education) or a control group (a traditional education) with a ratio of 1:1 using a random table. A different person from those recruiting and offering the education carried out the randomization. The investigators who executed the study and the occupational therapist and speech therapists who performed assessments were blinded to the allocation. However, children and their special education teacher were aware of their allocation.

### 2.3. Interventions

The intervention group underwent ‘touch screen-based cognitive training’ for 30 min/session and 3 times/week at special school, over 12 weeks instead of a traditional education. The frequency and duration of the intervention was decided according to the previous studies [4,5]. Since children had various cognitive function, the level of difficulty of the program was selected by the special education teacher, who helped the children using the program. 

The ‘touch screen-based cognitive training’ targets children with a cognitive age of 18–41 months. It was developed to target many different cognitive domains including attention, memory, visuospatial function, executive function, auditory cognition, language, and eye-hand coordination, with attention being the main target (Table 1). It has 12 programs in total. Six are adaptive programs and consist of 9 or 10 levels with various difficulties. The remaining 6 programs are non-adaptive and they have no levels of difficulty. The additional information can be found in a paper by Sung et al. [3].

The control group received a traditional education by special education teachers for 30 min/session. During the education, color matching, puzzles, blocks, identical image identification, tracing and finding hidden objects were used. The total education time was same in both groups.

### 2.4. Outcomes

Children were assessed by one occupational therapist and one speech therapist before and after the intervention. The outcomes evaluated general development, cognition, language, and ADL. The primary outcome was assessed using PEP-R, and secondary outcomes were: ECBQ [6], SELSI [7], Pediatric Evaluation of Disability Inventory (PEDI) [8], and Goal Attainment Scale (GAS) [9]. 

PEP-R [10] is a revised version of PEP, and includes a developmental scale (131 items) and a behavioral scale (43 items). The former consists of 7 scales, which are imitation, perception, fine motor activity, gross motor activity, eye–hand coordination, cognitive performance, and cognitive verbal operations. Adding all the individual item-passing scores of the developmental scale yields the developmental score and the standardized developmental age. In this study, developmental score and age were used as measurements of general development. ECBQ [6] assesses behavior between 18 and 36 months of age and is a parent-reported measurement. Among the 18 discrete traits of ECBQ, only the “attentional shifting” and “attentional focusing” traits were used in this study for the evaluation of attention. SELSI [7] assesses language abilities, receptive and expressive language age in children under 36 months old through the parent’s report. PEDI [8] measures independence in ADL in children (6 months–7.5 years), and consists of subscales of self-care, mobility, and social function. GAS measures an individual’s goal achievement and is a criterion-referenced measurement. GAS was first introduced in 5-point scale, but this study used 6-point scale [9]. Furthermore, data on the main diagnosis, sex, and age were collected.

### 2.5. Statistical Analysis

SPSS software for Windows version 20.0 (SPSS Inc., Chicago, IL, USA) was used to analyze the data, and *p*-values less than 0.05 were reported as statistically significant. When data were normally distributed, we used parametric statistics. When data were not normally distributed, we used nonparametric statistics. For comparing baseline characteristics, Chi-square test, Fisher’s exact test, and Mann–Whitney U test were used. The outcome measurements were analyzed for significant differences between the two groups using repeated measures analysis of variance (ANOVA), and independent variables of time, intervention and interactions were tested. Regression of post-education measurements in the group to adjust for the pre-education measurements was performed by the linear regression analysis: dependent variables were post-education outcome measurements, and independent variables were pre-education measurements and group. Several separate regression analyses were computed for each outcome measurement. Pearson’s correlation analysis was used when evaluating the efficacy of a ‘touch screen-based cognitive training’ in relation with the severeness of the developmental delay. The severeness of developmental delay was defined as the difference between the chronological age and the developmental age.

## 3. Results

### 3.1. Baseline Characteristics of the Children and Applicability of the ‘Touch Screen-Based Cognitive Training’ in Special Education

The intervention group included 17 children and the control group included 12 children (Table 2). The mean chronological age of the intervention group was 140.1 ± 49.1 months (approximately 11.7 years old), with 9 males and 8 females, 12 with ID, and 5 with ASD. The mean chronological age of the control group was 143.6 ± 60.3 months (approximately 11.9 years old), with 10 males and 2 females, 8 with ID, and 4 with ASD. The mean developmental age was 24.1 ± 8.5 months (approximately 2 years old) in the intervention group and 22.8 ± 6.5 months (approximately 1.9 years old) in the control group. There were no significant differences between the two groups in the measurements of baseline general development, cognition, language, and ADL.

All the children included in the intervention group were interested in the ‘touch screen-based cognitive training’, and they all completed 36 sessions of intervention without drop-out. They did not show any problems when performing the intervention (obsession with a tablet computer or irritable behavior).

### 3.2. Comparison of the Measurements within and between the Two Groups

Repeated measures ANOVA was used to compare the effect of time of intervention baseline to 12 weeks on outcome measurements, as shown in Table 3. Significant groupⅹtime effect was found for GAS, and significant group effect was found for ECBQ (attentional shifting) and GAS. There was significant time effect for most of the measurements. 

Since baseline measurements were better in the intervention group than the control group (Table 2), pre-education measurements were adjusted by using the linear regression analysis. The ‘touch screen-based cognitive training’ is shown to have a significant effect on the post-education measurements of ECBQ (attentional shifting) (β = −6.132; *p* = 0.029), and GAS (β = −1.083; *p* = 0.032) after adjusting for the pre-education measurements (Table 4).

### 3.3. The Efficacy of the ‘Touch Screen-Based Cognitive Training’ Considering the Severeness of Developmental Delay

There was no relationship between the changes in the measurements and the severeness of developmental delay in 17 children of the intervention group (Table 5). 

## 4. Discussion

This study indicates that the ‘touch screen-based cognitive training’ at a special school significantly improved cognition and individual goal achievement, as proven by attentional shifting of ECBQ and GAS. In repeated measures ANOVA, GAS was the only measurement to show groupⅹtime effect. In contrast, attentional shifting of ECBQ showed group effect, but not the groupⅹtime effect, indicating that the degree of improvement in the intervention group is not significantly different from the degree of improvement in the control group. However, when the pre-education measurements were adjusted, the ‘touch screen-based cognitive training’ had a meaningful impact on post-education ECBQ (attentional shifting) and GAS. In other words, if the baseline cognitive values were similar between the intervention and control groups, the intervention group will show better scores in the ECBQ (attentional shifting) and GAS than the control group. This improvement in the measure of attention is probably related with the characteristics of the intervention, which targets attention the most (Table 1). Furthermore, there was no relationship between the changes in the measurements and the severeness of developmental delay, which indicates that the degree of improvement is not affected by the severeness of developmental delay.

This result is somewhat different from a previous study [4], that showed improvements in social function of PEDI, observation and manipulation subscales of Laboratory Temperament Assessment Battery, and GAS when the intervention was used in hospital. These differences are probably due to the difference in chronological age (140.1 months vs. 54.8 months) which suggests that children in this study had more severe cognitive delay, and due to the difference in practitioner (special school teachers vs. occupational therapists).

Prior to the 1970s, children with disabilities were frequently refused enrollment or appropriately educated by public schools [11]. From the mid-1960s to 1975, the U.S. Congress, federal courts, and state legislatures spelled out strong educational rights for these children. When they are unable to acquire appropriate education in regular schools, special school should be considered to benefit the same educational opportunity [12]. In special schools, not only the academic curriculum, but also other developmental and supportive programs, such as physical therapy, cognitive therapy, occupational therapy, speech therapy, and behavior techniques are provided. Special schools try to provide individualized education, usually by teachers. However, there are some limitations in providing individualized education for every child, and the children have limited attention to give to participating in education, especially those with severe cognitive impairment. Therefore, other methods of education need to be explored.

Multimedia is a combination of different external representations, such as static or animated pictures, written or spoken texts, and sound [13]. Computer-based multimedia as a tool of education and learning has been tried in multiple previous studies [14,15,16]. Mayer and Sims [14] presented the potential of computer multimedia as teaching materials and showed that integration of pictures and texts showed improvement in learning. These findings can be explained with the Paivio’s dual coding theory [15]. In this theory, words are encoded by the verbal system, and pictures are encoded by both verbal and imagery systems. Therefore, pictures in texts enhance memory by using a dual coding system. Schnotz and Bannert [16] found that structure of graphics affects the structure of the mental model and asserted that only task-appropriate graphics enhance learning. This emphasizes the contents of graphics in learning mechanism.

There are few computer-based cognitive programs used in school setting. RoboMemo (a registered trademark of CogMed Cognitive Medical Systems AB, Stockholm, Sweden) [17] targets working memory in children 7–12 years of age, and there was a previous study showing the improvement of working memory and behavior after using this intervention in children with attention deficit hyperactivity disorder (ADHD) (*n* = 9, age 8–10.5 years) [18]. Braintrain is an attention training program, and it was effective in improving the parent rated inattentive behavior in children with ADHD (*n* = 35, mean age 12.4) [19]. Training Attention and Learning Initiative (TALI) trains attention by using a touch screen tablet, and it reduced inattention and hyperactivity in typically developing children (*n* = 98, age 5–9 years) [20].

There are some computer-based interventions targeting language for children to be used in school setting. Fast ForWord-Language targets literacy skills and oral language in children (4–14 years) who have difficulty in language learning. The intervention showed gains of 1–1.5 years on language in 7 children with language-learning impairment (age 5.9–9.1) [21]. Baldi is a computer-animated tutor, which teaches vocabulary and grammar, and it showed an improvement in the number of vocabulary words in children with ASD (*n* = 8, age 7–12) after the intervention [22]. The Alpha program is an interactive multimedia program developed for language learning through video, animation, voice, and sign language. It showed improved reading ability and language skills in children with ASD (mean age = 9.4 years), in children with mixed handicaps (mean age = 13.1), and in normal preschool children (mean age = 6.4 years) [23].

The ‘touch screen-based cognitive training’ used in this study is different from the interventions introduced above. First, this intervention targeted children below 4 years of cognitive age, whereas most of the interventions target children above 4 years of age. Second, it uses a tablet computer and touch screen, which is advantageous to the very young children and the children with severe cognitive delay. It has visual and auditory support systems, which can promote motivation of the children. Third, the interventions were very structured and standardized, enabling teachers to provide individualized programs. This randomized controlled trial showed that the ‘touch screen-based cognitive training’ is effective in children with severe cognitive impairment in special education. 

In COVID-19 era, schools were closed, and conventional education was stopped in many countries. Since there is a strong connection between education, earnings, and life expectancy [24], school closures may affect child health over the long them [25]. Therefore, some other form of education is required in this period. Since the ‘touch screen-based cognitive training’ decreases personal contacts, this might help children in education at low cost. Further, if it happens to be difficult to re-open schools, this intervention may be used at home because it is portable. By providing proper education in such children, improvements in cognition and active social participation can occur.

This study has some limitations. First, there was a limitation in the subjects enrolled. Small number of children were enrolled, and the rate of diagnosis (ID vs. ASD) was not equal in these children. In addition, there were more males than females in the Control group, although there were no significant differences. Furthermore, the etiology of the ID was not collected. Second, many schools and teachers participated in this study, which could introduce artifacts related to individual differences. To overcome this problem, the instructions for the ‘touch screen-based cognitive’ were offered to the teachers, to reduce the possible differences among them. Third, there are some limitations in the measurements. Only the developmental score and age of PEP-R were assessed in this study, not the developmental scales of 7 domains, limiting the further interpretation of the result. ECBQ was rated by each child’s special education teacher, who was not blinded to group allocation. The result of GAS should be interpreted cautiously because the goals are set individually according to the children’s priority. Furthermore, subjective satisfaction for the intervention was not assessed in this study. Lastly, long-term follow-up assessments should be considered in the future. 

## 5. Conclusions

Application of a ‘touch screen-based cognitive training’ in special school was feasible and it showed improvements in cognition in children with severe cognitive impairment of (developmental age 18–36 months), irrespective of the severeness of the developmental delay. These findings offer possibilities for the use of the intervention as an educational tool for children with cognitive impairment in special education. Furthermore, this intervention might give another option for the education for these children in the COVID-19 era.

## Figures and Tables

**Table 1:** Main cognitive targets of ‘touch screen-based cognitive training’.

	Main Cognitive Targets
**Adaptive programs**	
Puzzles	Attention, Visuospatial function, Language, Eye-hand coordination
Hidden object games	Attention, Memory, Visuospatial function, Executive function, Language
Animal matching	Attention, Visuospatial function, Executive function, Language
Pattern matching	Attention, Executive function
Identical image identification	Attention, Visuospatial function, Eye-hand coordination
Memory games	Attention, Memory, Executive function
**Non-adaptive programs**	
Tracing	Attention, Visuospatial function, Language, Eye-hand coordination
Object matching	Attention
Sound matching	Attention, Memory, Language, Auditory cognition
Balloon games	Attention, Memory, Visuospatial function, Language, Eye-hand coordination
Farm games	Attention
Daily activity games	Attention

**Table 2:** Comparison of baseline characteristics of children between intervention and control groups.

	Intervention Group (*n* = 17)	Control Group (*n* = 12)	*p*-Value
Age (months)	140.1 ± 49.1	143.6 ± 60.3	0.98
Sex (Male:Female)	9:8	10:2	0.13
Diagnosis (ID:ASD)	12:5	8:4	1.00
PEP-R (Developmental score)	61.6 ± 12.5	59.6 ± 9.6	0.66
PEP-R (Developmental age)	24.1 ± 8.5	22.8 ± 6.5	0.67
ECBQ (Attentional focusing)	41.1 ± 15.0	37.8 ± 7.3	0.49
ECBQ (Attentional shifting)	46.5 ± 12.4	35.2 ± 12.7	0.88
SELSI (Comprehension, raw score)	36.8 ± 13.4	34.3 ± 13.7	0.64
SELSI (Comprehension, age)	20.0 ± 7.1	18.6 ± 7.1	0.61
SELSI (Expression, raw score)	25.7 ± 13.1	19.2 ± 7.3	0.13
SELSI (Expression, age)	15.4 ± 8.2	11.4 ± 3.9	0.13
PEDI (Self-care)	47.0 ± 12.6	39.8 ± 16.3	0.13
PEDI (Mobility)	50.1 ± 13.2	47.1 ± 14.9	0.42
PEDI (Social function)	26.8 ± 6.2	24.7 ± 9.2	0.46
GAS	−2.0 ± 0.0	−2.0 ± 0.0	1.00

Values are presented as mean ± SD or number. ID, intellectual disability; ASD, autism spectrum disorder; PEP-R, Psychoeducational Profile-Revised; ECBQ, Early Childhood Behavior Questionnaire; SELSI, Sequenced Language Scale for Infants; PEDI, Pediatric Evaluation of Disability Inventory; GAS, Goal Attainment Scale. *p* > 0.05 by Chi-square test or Fisher’s exact test or Mann–Whitney U test.

**Table 3:** Comparison of measurements withing and between the two groups.

Variables	Time	Intervention Group (*n*=17)	Control Group (*n* = 12)	*p*-Value (Group × Time)	*p*-Value (Group)	*p*-Value (Time)
PEP-R (Developmental score)	Pre	61.6 ± 12.5	59.6 ± 9.6	0.07	0.30	<0.001 *
	Post	71.2 ± 13.7	63.5 ± 12.1			
PEP-R (Developmental age)	Pre	24.1 ± 8.5	22.8 ± 6.5	0.07	0.30	<0.001 *
	Post	30.7 ± 9.3	25.5 ± 8.1			
ECBQ (Attentional focusing)	Pre	41.1 ± 15.0	37.8 ± 7.3	0.16	0.31	0.003 *
	Post	45.5 ± 14.0	39.5 ± 7.8			
ECBQ (Attentional shifting)	Pre	46.5 ± 12.4	35.2 ± 12.7	0.88	0.005 *	0.004 *
	Post	52.0 ± 9.9	40.2 ± 7.2			
SELSI (Comprehension, raw score)	Pre	36.8 ± 13.4	34.3 ± 13.7	0.22	0.33	0.002 *
	Post	44.1 ± 9.3	37.7 ± 12.7			
SELSI (Comprehension, age)	Pre	20.0 ± 7.1	18.6 ± 7.1	0.21	0.29	0.003 *
	Post	23.8 ± 4.9	20.3 ± 6.5			
SELSI (Expression, raw score)	Pre	25.7 ± 13.1	19.2 ± 7.3	0.12	0.08	<0.001 *
	Post	30.6 ± 14.8	21.3 ± 7.6			
SELSI (Expression, age)	Pre	15.4 ± 8.2	11.4 ± 3.9	0.60	0.08	0.03 *
	Post	17.3 ± 7.8	12.6 ± 4.0			
PEDI (Self-care)	Pre	47.0 ± 12.6	39.8 ± 16.3	0.13	0.24	0.002 *
	Post	51.5 ± 13.1	43.0 ± 16.4			
PEDI (Mobility)	Pre	50.1 ± 13.2	47.1 ± 14.9	0.89	0.56	0.14
	Post	50.8 ± 13.4	47.7 ± 15.2			
PEDI (Social function)	Pre	26.8 ± 6.2	24.7 ± 9.2	0.13	0.24	0.002 *
	Post	30.7 ± 7.0	26.2 ± 8.5			
GAS	Pre	−2.0 ± 0.0	−2.0 ± 0.0	0.03 *	0.03 *	<0.001 *
	Post	0.0 ± 1.4	1.1 ± 1.1			

Values are presented as mean ± SD. PEP-R, Psychoeducational Profile-Revised; ECBQ, Early Childhood Behavior Questionnaire; SELSI, Sequenced Language Scale for Infants; PEDI, Pediatric Evaluation of Disability Inventory; GAS, Goal Attainment Scale. * *p* < 0.05 by the repeated measures ANOVA.

**Table 4:** The impact of the ‘touch screen-based cognitive training’ on the post-education measurements.

	Beta	SE	*p*-Value	95% CI
PEP-R (Developmental score)	−5.957	3.095	0.066	−12.344–0.430
PEP-R (Developmental age)	−4.045	2.093	0.065	−8.364–0.275
ECBQ (Attentional focusing)	−3.161	1.830	0.096	−6.923–0.601
ECBQ (Attentional shifting)	−6.132	2.649	0.029 *	−11.576–−0.687
SELSI (Comprehension, raw score)	−4.858	2.590	0.072	−10.192–0.475
SELSI (Comprehension, age)	−2.673	1.362	0.061	−5.477–0.132
SELSI (Expression, raw score)	−2.543	1.842	0.180	−6.388–1.251
SELSI (Expression, age)	−1.415	1.324	0.295	−4.141–1.311
PEDI (Self-care)	−1.822	2.279	0.431	−6.507–2.863
PEDI (Mobility)	−0.106	0.862	0.903	−1.879–1.666
PEDI (Social function)	−2.659	1.526	0.093	−5.797–0.478
GAS	−1.083	0.479	0.032 *	−2.068–−0.099

PEP-R, Psychoeducational Profile-Revised; ECBQ, Early Childhood Behavior Questionnaire; SELSI, Sequenced Language Scale for Infants; PEDI, Pediatric Evaluation of Disability Inventory; GAS, Goal Attainment Scale; SE, Standard Error; CI, Confidence interval. * *p* < 0.05 by linear regression analysis.

**Table 5:** Pearson’s correlation analysis between the changes in the measurements and the severeness of developmental delay (*n* = 17).

	*r*	*p*-Value
PEP-R (Developmental score)	−0.209	0.276
PEP-R (Developmental age)	−0.211	0.272
ECBQ (Attentional focusing)	−0.081	0.678
ECBQ (Attentional shifting)	−0.161	0.404
SELSI (Comprehension, raw score)	0.036	0.855
SELSI (Comprehension, age)	0.056	0.774
SELSI (Expression, raw score)	0.037	0.847
SELSI (Expression, age)	0.009	0.963
PEDI (Self-care)	−0.283	0.137
PEDI (Mobility)	0.065	0.739
PEDI (Social function)	−0.036	0.855
GAS	0.019	0.922

PEP-R, Psychoeducational Profile-Revised; ECBQ, Early Childhood Behavior Questionnaire; SELSI, Sequenced Language Scale for Infants; PEDI, Pediatric Evaluation of Disability Inventory; GAS, Goal Attainment Scale; *r,* correlation coefficient.

## Data Availability

Not applicable.
